# Dosimetric feasibility of carbon-ion radiotherapy for ocular melanomas with local effect model-based treatment planning system

**DOI:** 10.1093/jrr/rrag062

**Published:** 2026-07-31

**Authors:** Yuze Yang, Jiayao Sun, Xue Ming, Weixu Hu, Lin Kong, Yinxiangzi Sheng

**Affiliations:** Department of Medical Physics, Shanghai Proton and Heavy Ion Center, Fudan University Cancer Hospital, 4365 Kangxin Road, Pudong District, Shanghai 201315, China; Shanghai Key Laboratory of Radiation Oncology (20dz2261000), 4365 Kangxin Road, Pudong District, Shanghai 201315, China; Shanghai Engineering Research Center of Proton and Heavy Ion Radiation Therapy, 4365 Kangxin Road, Pudong District, Shanghai 201315, China; Department of Medical Physics, Shanghai Proton and Heavy Ion Center, Fudan University Cancer Hospital, 4365 Kangxin Road, Pudong District, Shanghai 201315, China; Shanghai Key Laboratory of Radiation Oncology (20dz2261000), 4365 Kangxin Road, Pudong District, Shanghai 201315, China; Shanghai Engineering Research Center of Proton and Heavy Ion Radiation Therapy, 4365 Kangxin Road, Pudong District, Shanghai 201315, China; Department of Medical Physics, Shanghai Proton and Heavy Ion Center, Fudan University Cancer Hospital, 4365 Kangxin Road, Pudong District, Shanghai 201315, China; Shanghai Key Laboratory of Radiation Oncology (20dz2261000), 4365 Kangxin Road, Pudong District, Shanghai 201315, China; Shanghai Engineering Research Center of Proton and Heavy Ion Radiation Therapy, 4365 Kangxin Road, Pudong District, Shanghai 201315, China; Shanghai Key Laboratory of Radiation Oncology (20dz2261000), 4365 Kangxin Road, Pudong District, Shanghai 201315, China; Shanghai Engineering Research Center of Proton and Heavy Ion Radiation Therapy, 4365 Kangxin Road, Pudong District, Shanghai 201315, China; Department of Radiation Oncology, Shanghai Proton and Heavy Ion Center, Fudan University Shanghai Cancer Center, 4365 Kangxin Road, Pudong District, Shanghai 201315, China; Shanghai Key Laboratory of Radiation Oncology (20dz2261000), 4365 Kangxin Road, Pudong District, Shanghai 201315, China; Shanghai Engineering Research Center of Proton and Heavy Ion Radiation Therapy, 4365 Kangxin Road, Pudong District, Shanghai 201315, China; Department of Radiation Oncology, Shanghai Proton and Heavy Ion Center, Fudan University Shanghai Cancer Center, 4365 Kangxin Road, Pudong District, Shanghai 201315, China; Department of Medical Physics, Shanghai Proton and Heavy Ion Center, Fudan University Cancer Hospital, 4365 Kangxin Road, Pudong District, Shanghai 201315, China; Shanghai Key Laboratory of Radiation Oncology (20dz2261000), 4365 Kangxin Road, Pudong District, Shanghai 201315, China; Shanghai Engineering Research Center of Proton and Heavy Ion Radiation Therapy, 4365 Kangxin Road, Pudong District, Shanghai 201315, China

**Keywords:** carbon ion, ocular melanoma, RBE-weighted dose, dose measurement

## Abstract

This study evaluates the dosimetric feasibility of carbon-ion radiation therapy (CIRT) for ocular melanoma (OM) using a local effect model-I–based treatment planning system (TPS) combined with pencil beam scanning delivery. Cube-shaped targets were designed based on the experience of CIRT for OM at the National Institutes for Quantum Science and Technology (QST). Treatment plans used the local effect model-I–based TPS (Syngo® V13C, SIEMENS, Germany) prescribing an absorbed dose consistent with values reported in the literature. A conversion factor (CF) was established to convert the QST dose to LEM based relative biological effectiveness (RBE)–weighted dose. The physical doses were verified by an in-house Monte Carlo program and measurement. The CF for the LEM, equivalent to the QST RBE-prescribed dose of 70 Gy (RBE) over 5 fractions, was established as 0.65. The 3D gamma passing rate between TPS and MC was ≥94% using 3%–3 mm criteria. Mean dose deviations between the TPS and measurement were ≤±1.61% for all targets sizes except the smallest one (7 mm). Mean distance-to-agreement was <1 mm for all plans except one with a range shifter (distance-to-agreement = 2.92 mm). Phantom cases showed a lateral penumbra of 5.4–6.2 mm, and distal dose fall-off measures 3.6 and 5.6 mm. The results underscore the need for RBE-weighted dose conversion when applying QST’s CIRT experience to OM treatment.

## INTRODUCTION

Ocular melanoma (OM) represents 5% of all melanomas with the eye being the second most common site for primary melanoma after the skin [1]. It is the most common intraocular tumor in the adult population [2], with its annual incidence rate being 5–6 cases per million people in Western and 0.2–0.6 cases per million people in Asian populations [3]. Although enucleation is sometimes used to remove OMs, a conservative approach using either radioactive plaques (brachytherapy) or external beams of radiation is often preferred.

Brachytherapy is suited to treat small to medium-sized OMs measuring <10 mm in thickness and <18 mm in diameter [4]. Ion beam radiotherapy using protons, helium ions and carbon ions have shown satisfactory results in the treatment of OM of all types [5–9]. Treatment of OM with proton beams as described in 1977 has resulted in a successful tumor control rate better than 90% [10]. Treatment of OM using a carbon-ion beam was first reported by the National Institutes for Quantum Science and Technology (QST, previously known as the National Institute of Radiological Sciences) and declared that carbon-ion radiation therapy (CIRT) provided outcomes comparable to those achieved with proton radiation therapy (PRT) [11]. A total of 206 OM patients were treated before July 2017 [12] resulting in a 3-year eye retention rate of 96.1% which was better than similar groups of patients using PRT [7]. They concluded that CIRT with CT-based treatment planning may be an alternative to PRT. The study by Aoki *et al*. [13] reported on the treatment of 250 patients with choroidal malignant melanoma using carbon-ion radiotherapy at the QST facility. The eye preservation rate (EPR) following CIRT was 93.2% at 5 years and remained above 90% at 8 years. This outcome is notably favorable when compared to the 5-year EPR after PRT, which ranges from 75.3% to 93.0%. The findings reinforce confidence in the use of CIRT for OM, suggesting that it is a promising treatment modality that not only controls tumors effectively but also preserves the eye and visual acuity.

One advantage of carbon-ion beams compared to X-ray or proton beams for cancer treatment could be their higher relative biological effectiveness (RBE) making them a good choice for hypofractionated radiotherapy [14]. Some treatment planning systems (TPSs) can calculate the RBE directly using one of several models developed for CIRT [15].

At QST, the RBE model for dose calculation initially adopted the HIMAC semi-empirical model as described by Kanai *et al*. [16] and was later transitioned to the modified microdosimetric kinetic model (m-MKM) [13, 17–19]. Although current QST clinical practice is based on m-MKM, the published clinical experience of carbon-ion radiotherapy for OM was established based on human malignant melanoma-I cells with a survival endpoint of 0.13% [20]. This framework is distinct from the Kanai model, which is based on human salivary gland cells with a 10% survival endpoint.

In contrast, the commercial TPS Syngo®, which is used in our center and in several heavy ion centers worldwide, employs the local effect model I (LEM-I) to convert physical dose into RBE-weighted dose. To enable consistent interpretation of CT-based ocular carbon-ion treatment plans and comparison with published QST clinical experience, it is therefore necessary to establish a quantitative relationship between LEM-based and HIMAC semi-empirical model–based biological dose estimates.

In addition, Magro *et al*. [21] reported that proton physical doses calculated by Syngo® differed significantly from measurements for small and shallow targets. Carbon-ion dose calculations may encounter analogous challenges. It was therefore also thought necessary to clarify whether Syngo® was sufficiently accurate for use with the small area, shallow penetrating carbon-ion beams used for OM treatments.

This study investigates the dosimetric feasibility of carbon-ion radiotherapy for OM using a LEM-I-based commercial TPS. The objectives of this work are twofold. First, we derive a conversion factor (CF) between the HIMAC semi-empirical model based RBE-weighted dose and the LEM-I-based RBE-weighted dose for OM. Second, we evaluate the accuracy of carbon-ion physical dose calculations for small and shallow targets by comparison with Monte Carlo (MC) simulations and experimental measurements. Subsequently, a new CT-based CIRT treatment protocol for OM was established.

## MATERIALS AND METHODS

### Treatment planning and RBE-weighted dose conversion

The commercial Syngo® TPS (V13C, Siemens, Germany) used in our clinic was commissioned and put into operation in 2014. For carbon-ion radiotherapy, Syngo® supports active beam delivery using a raster-scanning technique with energy stacking to generate Spread Out Bragg Peaks (SOBPs).

For the sake of clarity and consistency later in the text, the HIMAC semi-empirical model applied in QST’s OM treatment practices is referred to as the QST model or QST RBE-weighted dose. Tsuji *et al*. [11] reported the clinical application of QST model in OM treatment, prescribing a RBE-weighted dose of 70 Gy (RBE) delivered in 5 fractions and designing cube-shaped targets with a beam range of 38.3 mm (R38.3) and side lengths of 20, 25 and 30 mm (S20, S25, S30). The character “R” represents the target range while the character “S” represents the side length of the cube. For example, R38.3S20 represents a 20 × 20 × 20 mm^3^ cube target with a range of 38.3 mm in a water phantom. These clinical dose regimens and target parameter settings served as the core reference basis for the present study. To achieve the conversion from the QST biological dose to the LEM-I-based dose, we adopted a method similar to that reported by Fossati *et al*. [22]. The goal of this conversion method was to obtain physical dose distributions for the LEM-I that were consistent with those reported in Tsuji *et al*.’s [11] study. Based on preliminary data from published studies and pilot calculations, the approximate LEM-I-based RBE-weighted dose corresponding to the 70 Gy (RBE)/5 fractions QST RBE-weighted dose was estimated at 45 Gy (RBE) in total. A dose escalation range of 43.5–46.5 Gy (RBE) (5 fractions, 0.5 Gy (RBE) increments) was thus established to identify the matching dose with high precision. The one-dimensional physical dose profile of each Syngo® plan along the central axis within the target area was compared with the physical dose profile reported in the QST paper [11]. The plans with minimized root-mean-square (RMS) deviations of physical dose were used to identify the CF.

In the present study, all treatment plans were designed using scanned pencil beam delivery without the use of patient-specific apertures or compensators. Dose shaping in both the lateral and depth directions was achieved by magnetic beam scanning and energy modulation. All cube plans were optimized with a spot-to-spot lateral spacing of 1 mm and a range step of 3 mm, consistent with our clinically validated carbon beam model configured with a 3 mm ripple filter (RiFi) [23]. The full width of half maximum of carbon-ion spots in the air at the isocenter varied within this energy range between 6.9 and 9.6 mm, which means the ratio of spot size to inter-spot spacing was larger than 6:1. The water equivalent depth of the minimum carbon-ion energy in this facility is 20 mm; therefore, a 20 mm polymethyl methacrylate (PMMA) range shifter (RS) was applied for the shallow target (R38.3S30). The calculation grid of the physical dose and RBE-weighted dose was 1 × 1 × 1 mm^3^. Both the physical and RBE-weighted depth dose profiles of those cubes were compared with data reported in the literature. In addition, cube targets with a range of 38.3 mm (R38.3) and side lengths of 7, 10, 15 mm (S7, S10, S15) and were also generated for physical dose verification purposes. Treatment plans with 45 Gy (RBE) prescribed RBE-weighted doses were given to each of the target volumes using 5 fractions.

### Validation of physical dose through MC simulations

Quality assurance plans were generated in Syngo® by applying the treatment plans to a virtual water tank (WT) phantom. The WT phantom has a 5 mm thick PMMA front wall and a water volume of 30 × 40 × 40 cm^3^. Corresponding dose distributions were used for physical dose comparison.

In this work, the MC tool used for physical dose simulation was a track-repeating algorithm called the fast dose calculator (FDC) [24] which has been implemented into an *in-house* developed treatment information management and planning system (TIMPS®). The MC model of the scanning carbon-ion machine was created and validated using commissioning measurements. A portal quality assurance module in TIMPS® was utilized to simulate the physical dose distribution in the same virtual water phantom as used in Syngo®. A 3D Gamma analysis [25, 26] was performed to compare the physical doses calculated by Syngo® and TIMPS®. Voxels having doses below 5% of the maximum dose within the distribution were not used in the analysis. Multiple Gamma index criteria of 1% dose difference (DD) and 1 mm distance-to-agreement (DTA), 2% and 2 mm and 3% and 2 mm were selected for the analyses. A gamma passing rate (γ-PR) of ≥90% was defined as the clinical acceptance criterion for all gamma analysis criteria, consistent with the common practice for heavy-ion radiotherapy treatment planning verification.

### Verification of physical dose through measurements

For the measurements, 24 small thimble ionization chambers (PTW model 31 015, PTW, Germany) were placed in an array within the WT phantom described earlier using a PMMA block attached to a three-dimensional scanning arm [27]. The expected dose at each chamber location was provided by the TPS. For comparisons of calculations with measurements, a Gamma analysis was not performed but separate DD and DTA results between the calculated and measured doses of 24 chambers were analyzed. As in the plan calculation comparisons, chambers with measured doses below 5% of the maximum dose were not evaluated. A DD within ±3% and a DTA within 2 mm was defined as the clinically acceptable criteria for this verification. Detailed planning verification procedures can be found elsewhere [28].

In addition, depth-dose profiles were measured for the cube plans R38.3S20, R38.3S25 and R38.3S30, and a lateral dose profile was measured for R38.3S25 at central SOBP depth. Dose deviations between the calculated and measured responses were then evaluated and analyzed.

### Example of treatment plan dose distribution

A head phantom was CT scanned, and two target volumes representing common OM locations were manually contoured on the planning CT. Each target volume was approximately 1 cc. Using a 90° horizontal treatment beam angle, a carbon-ion treatment plan of 45 Gy (RBE) in 5 fractions was created to demonstrate target coverage along with the lateral penumbra and distal fall-off based on the LEM-I.

## RESULTS

### Conversion of RBE-weighted dose

A comprehensive table of RMS values corresponding to the LEM-I RBE-weighted doses ranging from 43.5 to 46.5 Gy (RBE) can be found in Table 1. The RMS deviations for the R38.3S20, R38.3S25 and R38.3S30 targets are minimized at 44.5, 45 and 45.5 Gy (RBE), respectively. The CF averaged over all target volumes for an QST model RBE-weighted dose of 70 Gy (RBE) was 0.64 ± 0.1. Physical and RBE-weighted depth dose profiles for single fractions of 14 Gy (RBE) QST model RBE-weighted dose of R38.3S20, R38.3S25 and R38.3S30 targets are shown in Fig. 1. Reference QST model physical and RBE-weighted dose profiles were derived from the profiles reported in fig. 2 of Ref. [11]. For all panels (A–C) in Fig. 1, a distinct observation is that the distal fall-off of both the physical dose (Phys_LEM) and RBE-weighted dose (Eff_LEM) calculated by Syngo® is more gradual, in contrast to the relatively steeper distal fall-off of the corresponding dose profiles (Phys_QST, Eff_QST) from the QST data.

**Table 1:** RMS deviation of physical dose profiles corresponding to QST model RBE dose of 70 Gy (RBE) and LEM model RBE doses of 43.5–46.5 Gy (RBE).

Target	QST model RBE dose (Gy (RBE))	LEM model RBE dose (Gy (RBE))	Physical dose profile RMS dev.
R38.3S20	70	43.5	0.31
		44	0.27
		**44.5**	**0.23**
		45	0.26
		45.5	0.35
		46	0.27
		46.5	0.34
R38.3S25	70	43.5	0.39
		44	0.33
		44.5	0.24
		**45**	**0.21**
		45.5	0.24
		46	0.25
		46.5	0.30
R38.3S30	70	43.5	0.39
		44	0.31
		44.5	0.27
		45	0.21
		**45.5**	**0.18**
		46	0.19
		46.5	0.23

Bold values indicate the prescribed doses at which the RMS deviations are minimized for the corresponding targets.

**Fig. 1:**
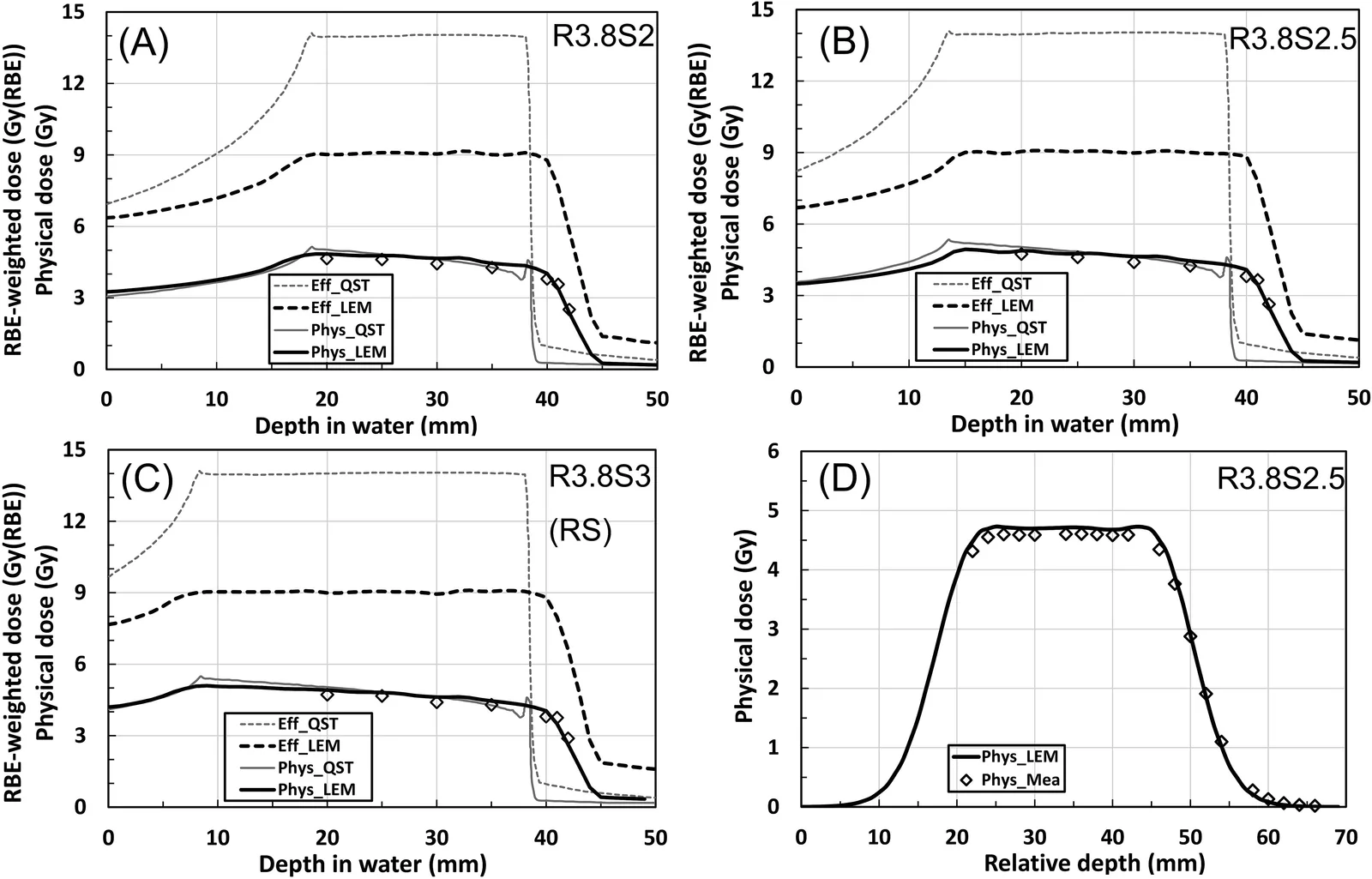
Single-fraction dose comparisons between the LEM and QST models for different targets: (A) R38.3S20, (B) and (D) R38.3S25 and (C) R38.3S30. The gray dashed curves represent the RBE-weighted doses for the QST model plans, and the black dashed curves represent the RBE-weighted doses for the LEM plans. The gray solid curves represent the physical doses for the QST plans, and the black solid curves represent the physical doses for the LEM plans. The black diamond symbols represent the physical doses measured in the WT using pinpoint chambers. For all targets, the prescribed QST model RBE-weighted dose was 14 Gy (RBE) per fraction for 5 fractions (data extracted from Tsuji *et al*. [11]).

### Validation of physical dose with MC simulations

The γ-PR with various criteria calculated from the MC simulations are shown in Table 2. Using criteria of 3%–3 mm, the γ-PRs for most cubes were higher than 97.5%. The lowest γ-PR was for the cube R38.3S30 which was 94.92%. The γ-PRs using criteria of 2%–2 mm for most cubes were higher than 97% except for the cube R38.3S30, which was 77.99%. The cube R38.3S30 with the lowest PR used an RS.

**Table 2:** Gamma passing rate for the physical dose of cube plans compared with Monte Carlo simulation

Target	LE model RBE-weighted dose (Gy (RBE))	γ-PR criteria		
		1%–1 mm (%)	2%–2 mm (%)	3%–3 mm (%)
R38.3S7	45	88.74	98.55	98.77
R38.3S10	45	87.08	98.34	98.58
R38.3S15	45	83.50	97.31	98.12
R38.3S20	44.5	83.11	97.40	97.99
R38.3S25	45	83.11	97.40	97.99
R38.3S30 (with RS)	45.5	60.70	77.99	94.92

### Validation of physical dose with measurements

Table 3 shows the results of the comparison of physical doses calculated by Syngo® with the measurements. Despite the small field sizes and ranges for the cube plans (with parameters similar to patient eye plans) and the spacing between detectors in the ion chamber array, the number of chambers receiving a dose of more than 5% of the maximum dose (*D*_max_) was always greater than or equal to 10 for all cubes except for the cubes R38.3S7 and R38.3S10. The calculated doses at the locations of those chambers varied from 0.28 to 4.85 Gy. The mean DD for each cube target varied from −0.28% ± 2.24% (mean ± standard deviation) to 1.61% ± 3.21% for all plans except for the cube R38.3S7 which had a mean dose deviation of 6.47%. The mean DTA showed agreement between the measured and planned doses to within 0.93 ± 1.28 mm for all cubes except for the cube R38.3S30 which had a mean distance of 2.92± 3.02 mm.

**Table 3:** Pinpoint chamber measurement of all cube plans

Targets	No. of points > 5% of *D* _max_	Calculated dose (Gy)			Measured dose (Gy)			DD (*D* _measure_ − *D* _cal_)/*D* _cal_		DTA (mm)	
		Mean	Min	Max	Mean	Min	Max	Mean (%)	SD (%)	Mean	SD
R38.3S7	5	2.02	0.96	3.94	2.28	1.01	3.86	6.47	18.07	0.93	1.28
R38.3S10	8	2.45	0.26	4.44	2.52	0.23	4.63	1.61	3.21	0.66	0.71
R38.3S15	10	2.75	0.60	4.68	2.72	0.45	4.57	−0.67	2.31	0.64	0.65
R38.3S20	11	2.95	0.28	4.84	2.90	0.24	4.76	−1.09	2.00	0.69	0.90
R38.3S25	11	3.31	0.55	4.85	3.29	0.50	4.76	−0.28	2.24	0.92	1.05
R38.3S30 (with RS)	14	2.90	0.28	4.76	2.84	0.20	4.59	−1.40	1.96	2.92	3.02

Figure 1 presents the measured and calculated depth-dose profiles for the cube plans R38.3S20, R38.3S25 and R38.3S30 (with RS), together with the lateral dose profile for R38.3S25. Appendix Table A1 summarizes the calculated and measured detector responses for the depth-dose profiles of R38.3S20, R38.3S25 and R38.3S30 (with RS), along with the corresponding dose deviations. Appendix Table A2 summarizes the lateral profile measurement for R38.3S25. The mean dose deviations relative to the calculated maximum dose were −0.68 ± 3.16%, −0.28 ± 3.06% and −0.55 ± 3.12% for the depth-dose profiles of R38.3S20, R38.3S25 and R38.3S30 (with RS), respectively, and −1.34 ± 1.72% for the lateral profile of R38.3S25. The maximum point dose deviation relative to the calculated maximum dose was 5.64%, observed at the distal edge of the depth-dose profile for R38.3S30 (with RS).

### Sample dose distributions and profiles

Figure 2 shows the dose distribution for two cases on the phantom. The 95% dose covers the target effectively. The lateral penumbra (from 80% to 20% of the prescribed dose) ranges from 5.4 to 6.2 mm. The distal dose falloff (from 80% to 20% of the prescribed dose) measures 3.6 and 5.6 mm for the two cases, respectively.

**Fig. 2:**
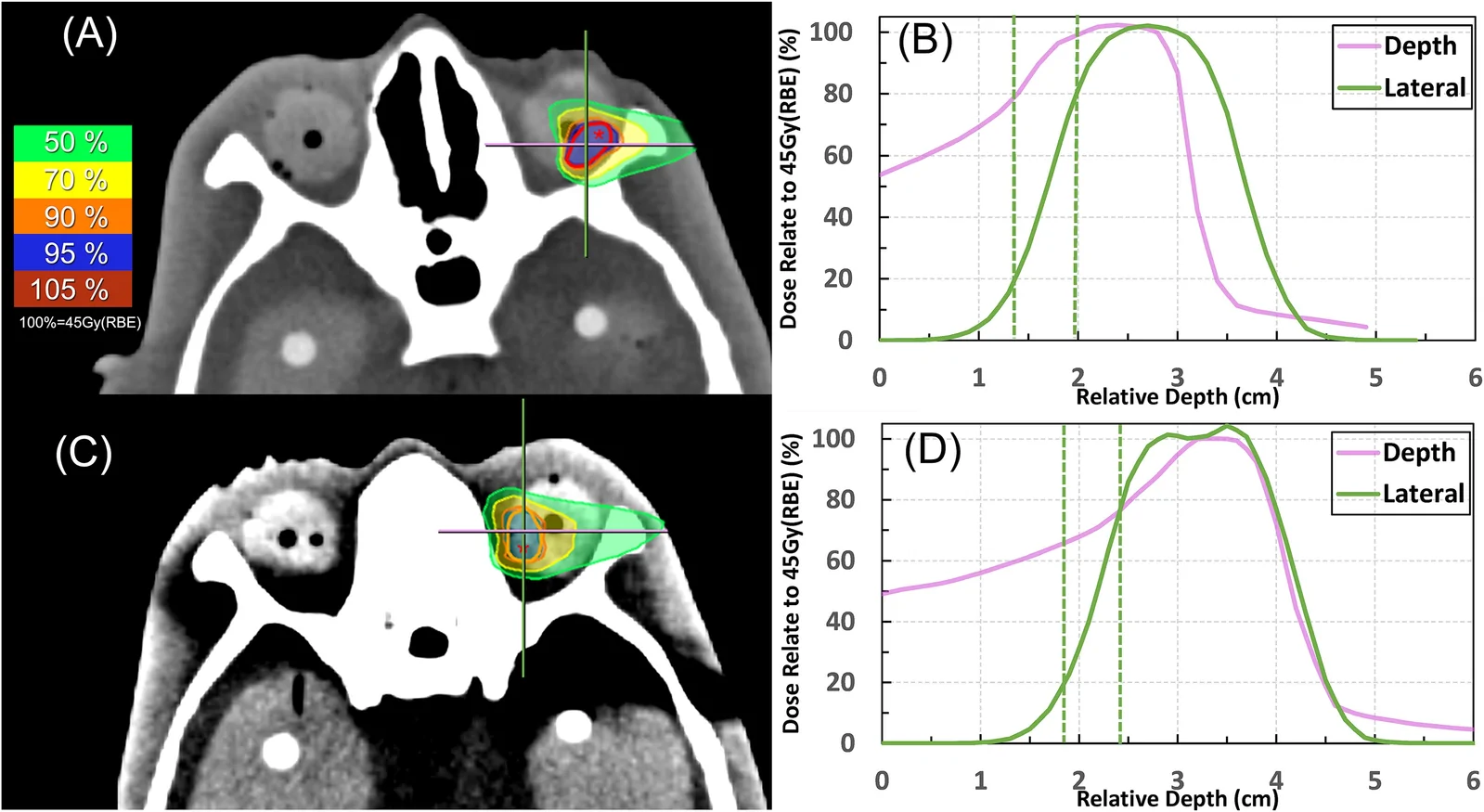
(A) and (C) two different cases dose distribution on phantom; (B) and (D) depth dose and lateral dose profiles of the two cases.

## DISCUSSION

CIRT has been considered for eye-conserving treatment of OM in Japan [7, 11], but the reported experience was limited to the use of the QST model-based RBE-weighted dose conversions and to scattered carbon-ion beams with apertures and physical range modulators for beam delivery. There has been no prior experience treating OM with an LEM-based RBE calculation TPS. The dosimetric feasibility, including the RBE-weighted dose and physical dose, merited special considerations for the OM CIRT due to high fractionated doses and small target volumes. In this study of OM CIRT, a CF was established to convert QST model doses used in previous treatments to LEM doses for use with the clinical TPS. In addition, the physical dose calculated by a commercial TPS was validated using both MC simulation and measurement.

Comparison and conversion of QST model RBE-weighted doses to LEM RBE-weighted doses was previously reported by Steinstrater *et al*. [29] who presented a method to convert clinically prescribed RBE-weighted doses from the QST model to both LEM-I and LEM-IV. Fossati *et al*. [22] had obtained the mean RBE-weighted doses with a MC code (FLUKA) and compared them to LEM-based prescription doses with the QST model dose by minimizing differences in the physical dose distributions between the two systems. Wang *et al*. [30] obtained conversion curves from prostate cancer patients that converted RBE-weighted doses inside and outside targets from QST model to LEM by using an experimental TPS (eTPS, V8A, Raystation, Sweden). The present study performed a direct comparison between QST model and LEM-based RBE-weighted dose and physical dose in cubes with small size and shallow location. Consequently, similar but more precise RBE-weighted dose CFs were obtained by comparing to previous publications and providing clinically useful data for LEM-based OM CIRT.

For all cube plans with or without RS, the physical dose distributions calculated by Syngo® passed the 3%–3 mm γ-test (taking MC simulation as reference). The mean dose deviation measured using pinpoint chambers was within ±1.61% except for the smallest target (R38.3S7 with target volume of 0.34 cc). These findings and the results reported by Magro *et al*. [21] support the ability of Syngo® to accurately calculate carbon-ion physical doses for shallow located target with ≥10 mm side length (or 1 cc volume).

By applying more restrictive criteria for the γ-test such as 2%–2 mm, the γ-PR is still acceptable for most plans except for the R38.3S30 cube plan which was generated with an RS. The mean DTA of this cube plan was 2.92 mm, while the other cube plans were all <1 mm. A mean DTA close to 3 mm is not acceptable in the context of clinical ocular particle therapy. The main reason is that Syngo® considers the RS in carbon-ion beams to be only an energy reducer without producing additional lateral scatter whereas the MC calculations in TIMPS consider both. This phenomenon has a relatively larger impact on small and shallow located targets. The shallowest depth that could be directly measured under our current experimental settings was 20 mm. Therefore, the dosimetric validation in this study should be considered applicable only to target regions located at depths of 20 mm or greater. When the commonly applied 2.5 mm margin in ocular radiotherapy is taken into account, the practical validated target depth is further restricted to approximately 22.5 mm or greater. This boundary is clinically relevant because an RS would be required for shallower targets; such plans should therefore be interpreted with caution and are not recommended under the current beam model and delivery configuration. One possible solution is to provide a more accurate description of beam transport and lateral spreading through the RS as recommended by Magro *et al*. [21]. Another easier solution could be to place a PMMA plate as close as possible to the patient and perform dose calculations with CT scan images that include the PMMA. However, this approach would require further evaluation and a strict geometric verification procedure before clinical implementation. The position, lateral alignment and air gap of the RS relative to the patient must be accurately reproduced between simulation and treatment.

One advantage of carbon-ion scanning beams compared to proton scanning beams without physical apertures is the smaller lateral penumbra at identical water equivalent depths. This advantage provides superior dose conformity to the targets compared with proton beams. Based on the results of this study, a method has been proposed to take advantage of the native carbon ion beam and perform CIRT for OM without apertures. The advantages of this proposal include:

- Use of a commercially available CT-based TPS which can provide optimization and accurate dose calculations for both target and organs at risk (OARs).
- Optimization of gantry angle or couch angle based on tumor location. The use of multiple beam angles per fraction is also possible.
- Only minor changes are required to start the CIRT treatment program for OM with an existing carbon ion beam facility.

The reference depth profiles shown in Fig. 1, reproduced from Ref. [11], were measured using a passive beam delivery with a patient-specific aperture. In contrast, the absence of an aperture in scanned beam delivery results in increased lateral dose spread, which may lead to higher dose exposure to OARs compared with proton or carbon-ion therapies employing apertures. Slopsema *et al*. [31] reported the lateral pernumbra at proton therapy centers for eye treatments typically between 1 and 2 mm (ranges from 0.9 to 2.2 mm), while the distal fall-off range from 0.7 to 6.6 mm. Rajesh *et al*. [32] reported an novel applicator on a conventional proton pencil beam scanning beamline. The lateral penumbra ranges from 2.6 to 4.4 mm for all range, modulation and depth combinations considered. The distal penumbra varied from 2.45 to 3.2 mm. In the case study presented in our research, the lateral pernumbra ranged from 5.4 to 6.2 mm, and distal fall-off was 3.6 and 5.6 mm. The relatively broad lateral penumbra and distal fall-off remain important limitations of the present approach for OM treatment, as they may reduce dose conformity and increase irradiation to surrounding normal tissues compared with aperture-based ocular irradiation techniques. Differences in distal dose fall-off were also observed when comparing the QST reference data, as shown in Fig. 1. These discrepancies arise primarily from differences in beam delivery and modulation strategies. The increased lateral penumbra observed in the present study can be attributed to the absence of an aperture. In proton ocular therapy, a predefined SOBP is typically generated based on the depth and width of the target using a range modulator. Similarly, in the QST reference data, beams are delivered using monoenergetic scattered beams, in which patient-specific compensators are employed to shape the distal edge and target geometry. As no longitudinal broadening of the Bragg peak is applied in either approach, the resulting physical dose profiles exhibit a very sharp distal fall-off. In contrast, the LEM-based plans in the present study were generated using a scanned beam delivery with energy stacking, where a 3-mm RiFi is used to longitudinally broaden individual Bragg peaks. While such broadening is generally negligible for deep-seated and larger targets, it becomes pronounced for ocular treatments characterized by very shallow ranges, with a modulation of approximately 38 mm in this study. It is anticipated that the use of a thinner ripple filter (e.g. 2 mm) or omitting the RiFi would reduce the distal fall-off to levels more comparable to those reported for proton ocular therapy and the QST reference data.

Although the lateral penumbra and distal falloff in the sample plan do not outperform the reported proton values, CT-based multi-field optimization may offer a more conformal dose distribution in the entrance directions compared with scatter-beam SOBP plans with fixed apertures, particularly for irregularly shaped target volumes. This concept is consistent with the study by Toyama *et al*. [7], in which CT-based two-port orthogonal carbon-ion therapy for choroidal melanoma was reported to reduce the incidence of neovascular glaucoma. Nevertheless, the practical benefit of multi-field planning depends on reliable OAR delineation, image registration and dose calculation. Further patient-specific verification and systematic evaluation of OAR-dose uncertainties across different tumor sizes and locations are still required.Another disadvantage is the small tail dose beyond the Bragg peak associated with carbon ion beams which are not present for proton beams. On the other hand, these shortcomings could be reduced by choosing more than one beam angle instead of using a single beam angle that enters through the anterior of the eye, typically used in proton plans.

The dose CF recommended in this study is based on the treatment of OM in 5 fractions as aforementioned. Despite the effort to match the physical dose, this conversion still has limitations. The physical dose could not match perfectly, especially for the regions near and beyond the distal edge of the target (Fig. 1). One of the main reasons was the 3 mm RiFi used in our center. It is recommended to conduct a prospective dose-escalation study to better clarify the effectiveness of the proposed method to treat OM by using a carbon ion beam.

## CONCLUSIONS

The dosimetric feasibility of treating OM using carbon ion beams with an LEM based RBE-weighted dose calculations was evaluated. A CF for translating RBE-weighted doses from the QST model to the LEM was established for OM cases, enabling comparison with published clinical experience. The carbon ion beam physical dose calculations for small and shallow located targets were shown to accurately match both MC simulations and measurements. It is concluded that large OM lesions with a side length of ≥10 mm (or a volume of ≥1 cc) can be treated using carbon-ion beams with CT-based plan optimization. However, treatment plans involving the use of a RS are not recommended under the current beam modeling and delivery configuration. Nevertheless, further work is recommended to be carried out such as evaluation and comparison of OARs dose.

## Supplementary Material

Appendix_Document_1_rrag062
