# Supplementary material for: Dosimetric feasibility of carbon-ion radiotherapy for ocular melanomas with local effect model-based treatment planning system
Source: J Radiat Res. 2026 Jul 31;67(5):746–53. doi: 10.1093/jrr/rrag062 (PMC13650713; doi:10.1093/jrr/rrag062)
Supplement: Supplementary file 1 [file appendix_document_1_rrag062.docx]

Table A1. Depth dose profile measurement for cube plans.

| Target | Depth(mm) | Calculated Detector response (Gy) | Measured Detector response (Gy) | DD  (D_measure_ - D_cal_) / D_cal,max_ |
| --- | --- | --- | --- | --- |
| R38.3S20 | 20 | 4.79 | 4.65 | -2.92% |
|  | 25 | 4.71 | 4.61 | -2.15% |
|  | 30 | 4.61 | 4.43 | -3.74% |
|  | 35 | 4.28 | 4.27 | -0.21% |
|  | 40 | 3.97 | 3.80 | -3.74% |
|  | 41 | 3.40 | 3.57 | 3.55% |
|  | 42 | 2.30 | 2.51 | 4.43% |
|  |  |  | Mean ± SD | -0.68% ± 3.16% |
| R38.3S25 | 20 | 4.87 | 4.74 | -2.57% |
|  | 25 | 4.68 | 4.60 | -1.48% |
|  | 30 | 4.53 | 4.40 | -2.69% |
|  | 35 | 4.23 | 4.24 | 0.29% |
|  | 40 | 3.99 | 3.81 | -3.76% |
|  | 41 | 3.50 | 3.65 | 3.23% |
|  | 42 | 2.39 | 2.64 | 5.04% |
|  |  |  | Mean ± SD | -0.28% ± 3.06% |
| R38.3S30 (with RS) | 20 | 4.86 | 4.72 | -2.80% |
|  | 25 | 4.75 | 4.67 | -1.75% |
|  | 30 | 4.54 | 4.40 | -2.76% |
|  | 35 | 4.33 | 4.29 | -0.76% |
|  | 40 | 3.99 | 3.80 | -3.75% |
|  | 41 | 3.64 | 3.75 | 2.31% |
|  | 42 | 2.61 | 2.88 | 5.64% |
|  |  |  | Mean ± SD | -0.55% ± 3.12% |

Table A2. Lateral dose profile measurement for cube plan

| Target | Lateral (mm) | Calculated Detector response (Gy) | Measured Detector response (Gy) | DD  (D_measure_ - D_cal_) / D_cal,max_ |
| --- | --- | --- | --- | --- |
| R38.3S25 | 22 | 4.44 | 4.31 | -2.71% |
|  | 24 | 4.68 | 4.55 | -2.64% |
|  | 26 | 4.69 | 4.60 | -2.02% |
|  | 28 | 4.67 | 4.59 | -1.66% |
|  | 30 | 4.66 | 4.59 | -1.60% |
|  | 34 | 4.68 | 4.60 | -1.53% |
|  | 36 | 4.68 | 4.61 | -1.56% |
|  | 38 | 4.66 | 4.60 | -1.28% |
|  | 40 | 4.64 | 4.58 | -1.13% |
|  | 42 | 4.67 | 4.59 | -1.60% |
|  | 46 | 4.57 | 4.34 | -4.75% |
|  | 48 | 4.00 | 3.76 | -5.09% |
|  | 50 | 3.03 | 2.88 | -3.26% |
|  | 52 | 1.94 | 1.91 | -0.72% |
|  | 54 | 1.06 | 1.10 | 0.87% |
|  | 58 | 0.22 | 0.28 | 1.34% |
|  | 60 | 0.09 | 0.13 | 0.96% |
|  | 62 | 0.03 | 0.06 | 0.66% |
|  | 64 | 0.01 | 0.03 | 0.49% |
|  | 66 | 0.00 | 0.02 | 0.34% |
|  |  |  | Mean ± SD | -1.34% ± 1.72% |
